# Specific LED-based red light photo-stimulation procedures improve overall sperm function and reproductive performance of boar ejaculates

**DOI:** 10.1038/srep22569

**Published:** 2016-03-02

**Authors:** Marc Yeste, Francesc Codony, Efrén Estrada, Miquel Lleonart, Sam Balasch, Alejandro Peña, Sergi Bonet, Joan E. Rodríguez-Gil

**Affiliations:** 1 Department of Animal Medicine and Surgery, Faculty of Veterinary Medicine, Autonomous University of Barcelona, E-08193 Bellaterra (Cerdanyola del Vallès), Barcelona, Spain; 2 Nuffield Department of Obstetrics and Gynaecology, University of Oxford, Level 3, Women’s Centre, John Radcliffe Hospital, Headington, Oxford OX3 9DU, United Kingdom; 3 GenIUL, Rambla de Sant Nebridi 22, E-08222 Terrassa, Spain; 4 Servicios Genéticos Porcinos, S.L., E-08150 Les Masies de Roda, Spain; 5 Biotechnology of Animal and Human Reproduction (TechnoSperm), Department of Biology, Institute of Food and Agricultural Technology, University of Girona, E-17003 Girona, Spain

## Abstract

The present study evaluated the effects of exposing liquid-stored boar semen to different red light LED regimens on sperm quality and reproductive performance. Of all of the tested photo-stimulation procedures, the best pattern consisted of 10 min light, 10 min rest and 10 min of further light (10-10-10 pattern). This pattern induced an intense and transient increase in the majority of motility parameters, without modifying sperm viability and acrosome integrity. While incubating non-photo-stimulated sperm at 37 °C for 90 min decreased all sperm quality parameters, this reduction was prevented when the previously-described light procedure was applied. This effect was concomitant with an increase in the percentage of sperm with high mitochondrial membrane potential. When sperm were subjected to ‘*in vitro*’ capacitation, photo-stimulation also increased the percentage of sperm with capacitation-like changes in membrane structure. On the other hand, treating commercial semen doses intended for artificial insemination with the 10-10-10 photo-stimulation pattern significantly increased farrowing rates and the number of both total and live-born piglets for parturition. Therefore, our results indicate that a precise photo-stimulation procedure is able to increase the fertilising ability of boar sperm via a mechanism that could be related to mitochondrial function.

Artificial insemination (AI) with liquid-stored semen is currently the most used technique for reproductive management of pig farms in Western countries^1^,^2^. The widespread utilisation of AI is based upon its great efficiency, combined with the feasibility of its application and the reasonable, low economic cost. Despite these indisputable advantages, some aspects of AI may still be improved. In this context, it is worth mentioning that boar sperm quality relies on both intrinsic and extrinsic factors, which ultimately impacts upon reproductive performance. Intrinsic factors include age^3^, breed^4^ and testicular size^5^. Extrinsic variables mainly refer to sperm handling^6^, husbandry factors, such as nutrition^7^,^8^, social environment^9^ and rhythm of semen collection^10^, and environmental conditions, such as season^11^,^12^,^13^, temperature and photoperiod^7^,^14^,^15^, as a noticeable drop in reproductive performance can be detected from late-summer onwards in farms located in different climate areas^12^,^13^,^16^,^17^. Exposition time to light does not seem to be the main cause of this phenomenon, since daily variations of light, either natural or artificial, only affect boar semen production in the most extreme cases, when animals are in the dark^18^.

Variations in reproductive performance could be partially overridden through photo-stimulation of sperm samples prior to AI. Previous works in the last few years have found that the application of laser light-beams at low energy (wavelength ranging from 530 nm to 830 nm) induces an increase in motion parameters and in ATP content in mouse, human, dog, bull, sheep and rabbit sperm (See ref. 19 for review). Similar effects with incoherent light, i.e. visible light at a wavelength range of 400 nm–800 nm, have also been observed in mouse, human, bull and ram sperm^20^,^21^,^22^,^23^. Light effects on motility are concomitant with an increase in the sperm’s ability to withstand thermal stress^24^. At least in dogs, effects upon sperm were dependent on light energy^25^. Furthermore, photo-stimulation has also been reported to improve not only the resistance to cooled storage of rabbit and turkey sperm^26^,^27^, but also the sperm cryotolerance in poultry species such as chicken, turkey and pheasant^26^,^28^. Additionally, exposing sperm to red light (wavelength: 660 nm) also increases the ‘*in vivo*’ fertilising ability of ram sperm^22^.

Despite all of these results, the effects of photo-stimulation on boar sperm function are yet to be studied. Moreover, and from a practical perspective, laser application requires specific and often costly material that cannot be afforded by a commercial farm in ordinary conditions. Thus, the possibility of using other cheaper, light-stimulating systems that may have similar efficiency to laser devices deserves further attention. In this context, the present work tested the effects of exposing boar sperm to specific, red light emission diode (LED) on quality parameters and reproductive performance. A LED-based system was tested because it is cheaper and easier to maintain and utilise than are lasers and also possesses high photonic efficiency. The present study not only evaluated the effects of different red light photo-stimulation procedures on sperm quality parameters, but also on their ability to withstand thermal stress induced by that incubation. The light regimen yielding the best results ‘*in vitro*’ was subsequently utilised in a standard AI procedure in a commercial farm to test the effects of photo-stimulation upon reproductive performance.

## Results

### Effects of separate red light exposure procedures on sperm parameters following incubation at 37 °C for 90 min

**Figure 1:**
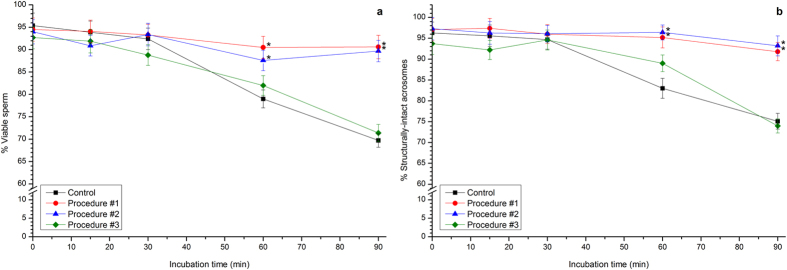
Percentages of viability and spermatozoa structurally-intact acrosomes of boar spermatozoa subjected to incubation at 37 °C for 90 min after photo-stimulation procedures. Boar sperm were subjected to separate photo-stimulation procedures and subsequent incubation at 37 °C as described in the Methods section. At the indicated times, aliquots were taken and percentages of viability (**a**) and structurally intact acrosomes (**b**) were determined. Results are shown as mean ± SEM. for 12 separate experiments. Asterisks indicate significant (*P* < 0.05) differences when compared with the respective Control value at the same time point.

Incubation of boar spermatozoa in a commercial extender at 37 °C induced a significant (*P* < 0.05) decrease of sperm viability, which went from 95.4% ± 2.8% at the start of the procedure to 69.7% ± 1.5% after 90 min (Fig. 1a). However, previous photo-stimulation of sperm samples with Procedure #1 significantly (*P* < 0.05) prevented that decrease, such that sperm viability in samples subjected to Procedure #1 was 90.6% ± 2.6% after 90 min of incubation at 37 °C. A similar effect was observed in samples subjected to Photo-stimulation Procedure #2 (Fig. 1a). On the contrary, Procedure #3 failed to exert any counteracting effect on the time-related decrease of sperm viability following incubation at 37 °C (Fig. 1a).

Percentages of acrosome-intact sperm also decreased when samples were incubated at 37 °C for 90 min (0 min: 96.3% ± 2.6% *vs.* 90 min: 75.1% ± 1.9%; Fig. 1b). Again, Procedures #1 and #2 counteracted that time-dependent decrease, and Procedure #3 had no effect (Fig. 1b).

**Figure 2:**
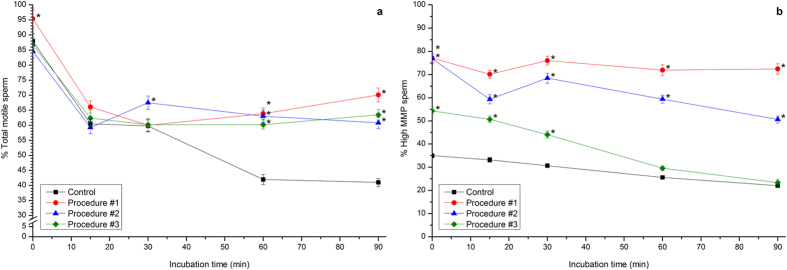
Percentages of total motility and high MMP spermatozoa of boar sperm cells subjected to incubation at 37 °C for 90 min after photo-stimulation procedures. Boar sperm were subjected to separate photo-stimulation procedures and subsequent incubation at 37 °C as described in the Methods section. At the indicated times, aliquots were taken and percentages of total motility (**a**) and high MMP sperm (**b**) were determined. Results are shown as mean ± SEM for 12 separate experiments. Asterisks indicate significant (*P* < 0.05) differences when compared with the respective Control value at the same time point.

Sperm motility also decreased following incubation at 37 °C (total sperm motility: 0 min: 88.0% ± 2.5% *vs.* 90 min: 41.0% ± 1.4%; Fig. 2a). Interestingly, Procedure #1, but not Procedures #2 and #3, induced a significant (*P* < 0.05), transient increase of total motility at the start of the experiment (Fig. 2a). A transient increase was also observed in Procedure #2 at 30 min. In addition to this, Procedures #1, #2 and #3 prevented the decrease in total motility observed after 60 min and 90 min of incubation in control sperm (Fig. 2a).

With regard to sperm kinetic parameters, controls incubated at 37 °C for 90 min showed a progressive decrease of VCL, which went from 62.6 μm·s^−1^ ± 3.4 μm·s^−1^ at 0 min of incubation to 42.3 μm·s^−1^ ±  2.0 μm·s^−1^ after 90 min of incubation (Table 1). Photo-stimulation Procedure #1 induced an immediate decrease of VCL values, which reached values of 49.6 μm·s^−1^  ±  2.3 μm·s^−1^ immediately after stimulation. However, this VCL-decrease later recovered, and VCL in Procedures #1, #2 and #3 was significantly (*P* < 0.05) higher than were control samples after 90 min of incubation (Table 1). In a similar fashion to that observed for VCL, Procedure #1 induced a significant (*P* < 0.05) decrease in VAP and ALH immediately after its application (Table 1). Additionally, after 90 min, VAP, STR and ALH in all three procedures, and VSL, LIN and BCF in Procedures #1 and #2 were significantly higher than in control (Table 1).

Finally, the percentage of boar sperm with high MMP in control samples was 35.0% ± 0.9% at 0 min of incubation and decreased progressively, reaching values of 22.1% ± 0.4% after 90 min of incubation (Fig. 2b). Photo-stimulation Procedure #1 induced an immediate increase in the percentage of cells with high MMP, which reached values of 77.1% ± 1.8% at 0 min of incubation. This percentage was roughly maintained during all of the incubation period, with values of 72.4% ± 2.3% after 90 min of incubation. While Photo-stimulation #2 showed a significant, although a much less intense, effect than did Procedure #1 (90 min: 50.6% ± 1.5%), Procedure #3 had no impact when compared to control samples (Fig. 2b).

### Effects of separate red light exposure procedures on the achievement of ‘*in vitro*’ capacitation and subsequent progesterone-induced acrosome exocytosis

**Figure 3:**
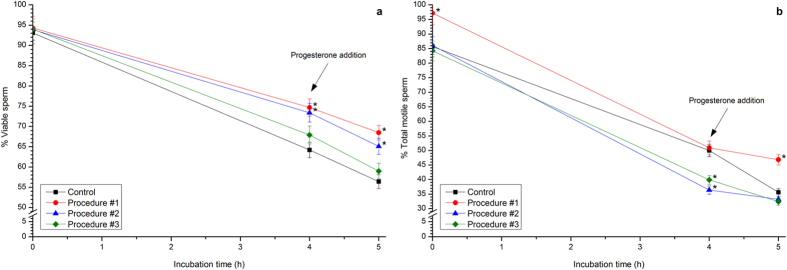
Percentages of viability and total motility of boar sperm cells subjected to ‘*in vitro*’ capacitation and subsequent progesterone-induced ‘*in vitro*’ acrosome exocytosis after photo-stimulation procedures. Boar sperm were subjected to separate photo-stimulation procedures and the subsequent IVC/IVAE as described in the Methods section. At the indicated times, aliquots were taken and percentages of viability (**a**) and total motility (**b**) were determined. 0: values at 0 h of incubation in the capacitation medium (CM). 4: values at 4 h of incubation in the CM. 5: values after 4 h IVC plus 60 min of the progesterone-induced IVAE. Results are shown as mean ± SEM for 12 separate experiments. Asterisks indicate significant (*P* < 0.05) differences when compared with the respective Control value at the same time point.

Incubation of control sperm with CM induced a significant (*P* < 0.05) decrease of viability, which went from 93.1% ± 2.7% at 0 h to 64.2% ± 1.9% after 4 h of incubation (Fig. 3a). A significant decrease in sperm viability was also observed following addition of progesterone and further incubation for 60 min (56.4% ± 1.7%). Interestingly, the decrease in sperm viability following IVC and IVAE was less intense when samples were subjected to Procedure #1 (74.7% ± 2.1% after 4 h of incubation in CM, and 68.5% ± 1.8% after 60 min of progesterone addition; Fig. 3a). Whereas the extent of effects induced by Procedure #2 was lower than by that of Procedure #1, Procedure #3 had no impact (Fig. 3a).

Incubation of control sperm with CM also induced a time-dependent decrease on sperm motility. Indeed, as shown in Fig. 3b, total sperm motility decreased in control cells from 85.7% ± 3.5% at 0 h to 50.0% ± 2.1% after 4 h of incubation, and to 35.6% ± 1.3% after 60 min of progesterone addition. Whereas total motility of sperm subjected to Procedure #1 was significantly higher (*P* < 0.05) than in control at 0 h (97.2% ± 3.9%; Fig. 3b), no significant differences were seen after 4 h of incubation. Notwithstanding, the decreasing rate of total sperm motility following progesterone addition was less intense in cells subjected to Procedure #1 than in control sperm (46.8% ± 1.8% after 60 min of progesterone addition; Fig. 3b). Procedures #2 and #3 did not affect total sperm motility at 0 h, and showed significantly (*P* < 0.05) lower values of this parameter than did control 4 h after incubation (Fig. 3b). These differences of these two procedures, with regard to control, were not observed 60 min after progesterone addition (Fig. 3b).

Regarding kinetic parameters, VCL, VSL, VAP, LIN, STR, WOB and BCF significantly (*P* < 0.05) increased in control sperm after incubation with CM for 4 h (Table 2). These increases were compatible with a feasible achievement of capacitation status, as previously described for boar sperm^29^,^30^,^31^. The addition of progesterone after 4 h of incubation induced a further increase in VCL, STR and ALH (Table 2). Procedure #1 significantly affected kinetic parameters following incubation with CM medium. Indeed, VCL, LIN and ALH were significantly (*P* < 0.05) higher than was control at 0 h and 4 h. Sixty minutes after progesterone addition, VCL, VSL, VAP, LIN, WOB and BCF in sperm photo-stimulated with Procedure #1 were significantly (*P* < 0.05) higher than in control sperm. In contrast, ALH in Procedure #1 was significantly lower than control 60 min after progesterone addition (Table 2). On the other hand, the impact of Procedure #2 upon sperm kinetic parameters was of a lesser extent than was that of Procedure #1, and only VCL showed significantly (*P* < 0.05) higher values than did control sperm. Finally, values of all kinetic parameters (except for BCF) in sperm subjected to Procedure #3 were significantly lower than were those of control samples both at 4 h and 60 min after progesterone addition (Table 2).

**Figure 4:**
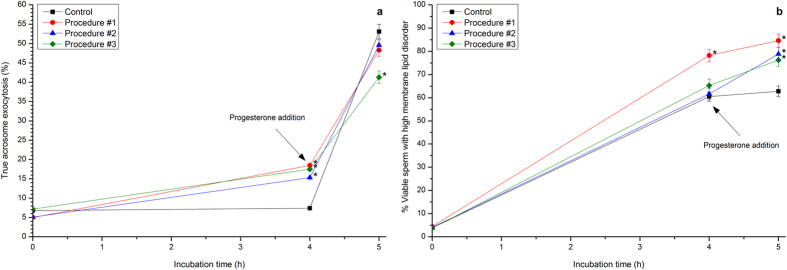
Percentages of true acrosome exocytosis and viable spermatozoa exhibiting high membrane lipid disorder following ‘*in vitro*’ capacitation and subsequent progesterone-induced ‘*in vitro*’ acrosome exocytosis after photo-stimulation procedures. Boar sperm were subjected to separate photo-stimulation procedures and the subsequent IVC/IVAE as described in the Methods section. At the indicated times, aliquots were taken and percentages of true acrosome exocytosis (**a**) and of those viable sperm exhibiting high membrane lipid disorder (**b**; percentages of M-540^+^-sperm are shown considering viable sperm only) were determined. 0: values at 0 h of incubation in the capacitation medium (CM). 4: values at 4 h of incubation in the CM. 5: values after 4 h IVC plus 60 min of the progesterone-induced IVAE. Results are shown as mean ± SEM for 12 separate experiments. Asterisks indicate significant (*P* < 0.05) differences when compared with the respective Control value at the same time point.

As expected, values of true acrosome exocytosis were very low in control samples incubated in CM. Those values only increased upon the addition of progesterone (53.1% ± 1.9% after 60 min of progesterone addition). No procedure affected values of true acrosome exocytosis at 0 h, but those values were significantly (*P* < 0.05) higher than in control sperm after 4 h of incubation in CM (e.g., 18.5% ± 0.2% in cells subjected to Procedure #1; Fig. 4a). After 60 min of progesterone addition, there were no differences between control and Procedures #1 and #2, and the percentages of true acrosome exocytosis were significantly (*P* < 0.05) lower in Procedure #3 than in the other treatments (41.3% ± 1.6%; Fig. 4a).

In control samples, the proportions of viable spermatozoa exhibiting high membrane lipid disorder increased along incubation with CM, reaching values of 60.5% ± 2.1% at 4 h (values are given considering viable sperm only; Fig. 4b). Interestingly, Procedure #1 induced a significant increase in the percentage of those viable sperm that exhibited high membrane lipid disorder, which reached values of 78.2% ± 2.7% at 4 h. This increase was not detected in sperm subjected to Procedures #2 and #3. In spite of this, all three photo-stimulation protocols induced a significant (*P* < 0.05) increase in the percentage of viable M540^+^-sperm after 60 min of progesterone addition (e.g., Procedure #1: 84.6% ± 2.9%; Fig. 4b).

**Figure 5:**
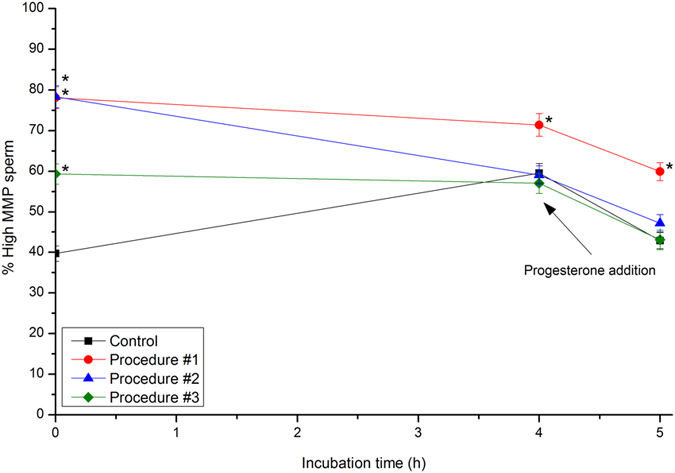
Percentages of sperm with high mitochondrial membrane potential (MMP; JC-1^+^). Boar sperm were subjected to separate photo-stimulation procedures and the subsequent IVC/IVAE as described in the Methods section. 0: values at 0 h of incubation in the capacitation medium (CM). 4: values at 4 h of incubation in the CM. 5: values after 4 h IVC plus 60 min of the progesterone-induced IVAE. Results are shown as mean ± SEM for 12 separate experiments. Asterisks indicate significant (*P* < 0.05) differences when compared with the respective Control value at the same time point.

Finally, incubation of control sperm with CM significantly (*P* < 0.05) increased percentages of sperm with high MMP, which reached values of 59.5% ± 2.4% at 4 h, which later decreased after 60 min of progesterone addition (42.9% ± 2.1%; Fig. 5). Procedure #1 dramatically affected this parameter, since there was an immediate and significant (*P* < 0.05) increase at 0 h (78.1% ± 2.7%). This increase was maintained throughout all of the experimental period, including incubation following progesterone addition (Fig. 5). While, before progesterone addition, the effects of Procedure #2 were similar, but less intense, than those induced by Procedure #1, the impact of Procedure #3 was only apparent at 0 h (Fig. 5).

### Effects of photo-stimulation upon reproductive performance of boar sperm

As shown in Table 3, treating seminal doses with Procedure #1 immediately before AI induced a clear and significant (*P* < 0.05) increase in FR (control: 83.7% *vs.* Procedure #1: 88.1%). Litter sizes were also significantly (*P* < 0.05) increased by photo-stimulation, since TP increased from 13.5 (control) to 14.9 (Procedure #1) and LP went from 12.7 (control) to 14.0 (Procedure #1). In all cases, LP piglets did not show any physiological or developmental alteration. Likewise, sows inseminated with photo-stimulated semen doses did not show any apparent differences in both their productive indexes and physiological and ethological characteristics during the gestation, farrowing and lactation periods, when compared with animals inseminated with non-photo-stimulated AI doses.

## Discussion

Our results indicate that a specific, red LED-based photo-stimulation procedure (Procedure #1) is able to increase the whole boar sperm response to both the heat stress due to incubation at 37 °C for 90 min and the achievement of IVC and subsequent, progesterone-induced IVAE. Moreover, the same photo-stimulation procedure is also able to significantly improve the ‘*in vivo*’ reproductive performance of liquid-stored boar semen. Although more in-depth information is still required, our results seem to indicate that the increase of heat resistance, the improvement of IVC achievement and the concomitant augment of ‘*in vivo*’ reproductive performance due to sperm photo-stimulation are driven by the same mechanism/s.

An important point that arises from our results is that the red LED-based photo-stimulation effects observed rely upon the specific pattern used. In this way, it is worth noting that Procedure #1 (10-10-10; L-phase: 10 min, D-phase: 10 min and L-phase: 10 min) was the most effective. In contrast, patterns with longer exposure times to light, such as Procedures #2 (15-10-15) and #3 (20-10-20) had less effect. Additionally, our preliminary experiments conducted before setting the experimental conditions also showed that continuous light-exposure patterns without a D-phase, of 5 min, 10 min, 15 min and 20 min of continuous L-phase, were much less effective than the 10-10-10 photo-stimulation pattern. These data clearly point out that the improving effect on boar sperm function induced by red LED-based light depends on the photo-stimulation pattern. A similar phenomenon has been described when laser systems are applied to sperm from other mammalian species like dog, buffalo and human^25^,^32^,^33^. Therefore, it seems that light-effects on mammalian sperm rely on precise rhythms and rates of application, regardless of light source and wavelength range.

At this moment, there is no clear explanation for this phenomenon, since the exact mechanism/s by which red light stimulates sperm is largely unknown. Despite this lack of information, a mechanism linked with light-induced activation of sperm mitochondria seems to be important to explain the effects observed. Indeed, our data regarding JC-1 analysis agree with this possibility. Taking this hypothesis as a basis, it could be suggested that different intensity and time of exposure would differently modify the activity of the whole mitochondria following interaction of light with elements of the mitochondrial electronic chain, such as photo-sensitive cytochrome complexes. In fact, this hypothesis would be in agreement with the results published by other authors, since cytochrome C/cytochrome C oxidase complex are activated by light wavelengths in the range of red-to-infrared^34^. It should be noted that the wavelength ranges utilised in the present work are able to activate the cytochrome C/cytochrome C oxidase complex. On the other hand, Passarella *et al*.^35^ observed that exposure of isolated mitochondria to irradiation with a He-Ne laser at an energy density of 5 J·cm^−2^ induced an increase in proton electrochemical potential and ATP synthesis. According to those authors, the laser-induced increase in ATP synthesis was due to laser-induced protomotive force. Furthermore, mitochondrial activation does not seem to be the only mechanism involved in response to photo-stimulation. Rather, photo-stimulation could also affect the utilisation of fructose through both glycolysis and oxidative phosphorylation, together with a simultaneous increase in the cell-uptake rates of phosphorus and calcium^36^. In fact, the glycolysis rate and both phosphorus and calcium uptake are indirectly modulated by mitochondria^37^,^38^,^39^. Nevertheless, without further research on this point, it is difficult to establish the actual relationship between sperm photo-stimulation and mitochondria-influenced variations of the glycolytic rate and phosphorus and calcium uptake.

Another hypothesis, previously established by Zan-Bar *et al*.^22^, would suggest that photo-stimulation could stimulate the production of reactive oxygen species (ROS). In this regard, it is worth noting that ROS are mainly synthesised in mitochondria as a product of the electronic chain^40^, and such molecules, together with reactive nitrogen species, are involved in the achievement of hyperactivation and acrosome exocytosis^41^,^42^,^43^,^44^. The most important proteins forming the mitochondrial electronic chain are those constituted in separate cytochrome/cytochrome oxidase structures^45^. This is important, since photo-stimulation at different wavelengths ultimately affects the activities of electronic-chain components, such as cytochrome C oxidase^34^. The activation of cytochrome systems could lead to an increase in the activity of the electronic chain, with concomitant effects on other processes, such as oxidative phosphorylation, ROS formation and, perhaps, activation of apoptotic-like signals linked to the achievement of sperm capacitation^34^. While the present study has not determined either the electronic-chain rhythm or ROS levels, it is worth noting that the ROS rate production in boar sperm is lower than in other species such as bovine or human^46^. Moreover, there is only a marginal increase in intracellular ROS levels of boar sperm following freeze-thawing, and this is again in contrast with human and bovine species^46^,^47^,^48^. This relatively low rate of ROS production in boar spermatozoa seems to be related to the specific functioning of mitochondria in these cells, which are in an uncoupled status during the majority of their lifespan^49^. This hypothesis could also explain our data in the achievement of both IVC and progesterone-induced IVAE. Thus, our results seem to indicate that Photo-stimulation Procedure #1 accelerates the achievement of IVC through mechanism/s concomitant with a significant increase in the overall mitochondrial activity, as JC-1 data suggest. All of these findings, especially the effects upon mitochondrial membrane potential reported herein, warrant further research on the electronic chain rate and ROS production following photo-stimulation not only in pigs but also in other mammalian species.

Despite all of the aforementioned data, the most interesting finding of this work was that Photo-stimulation Procedure #1 was able to increase ‘*in vivo*’ reproductive performance, evaluated through farrowing rates and litter sizes. Furthermore, that photo-stimulation had no deleterious effects on the piglets obtained, since there were no increases either in the number of stillborn animals or in the number of piglets born alive with physiological and/or structural alterations. In this way, we can conclude that the sperm photo-stimulation procedure is of such an innocuous nature that there are no impairing effects on the resulting offspring. The climatic conditions in which our ‘*in vivo*’ fertility assays were carried out did not by themselves affect the reproductive performance of the farm, and control sows were simultaneously inseminated. Since the overall fertility of the farm was maintained at a high level in control conditions, it was difficult to obtain greater quantitative differences between control animals and those inseminated with photo-stimulated semen. In spite of this, our data can be considered as conclusive since the number of inseminated sows is high, which results in significant statistical differences between the two groups.

While a similar increase in ‘*in vivo*’ fertility data has been observed in other livestock species, such as rams^22^, to the best of our knowledge there are no previous studies about the effects of sperm photo-stimulation on prolificacy. In our case, the prolificacy increase observed could be due to a photo-stimulation-linked increase in the ‘*in vivo*’ fertilising ability of boar sperm. Following this hypothesis, photo-stimulated spermatozoa would be more adapted to survive in the oviductal environment until the moment of fertilisation. This increase would match with our results obtained in both thermal resistance and IVC/IVAE experiments. In summary, photo-stimulation improves the boar sperm response to phenomena like the achievement of a feasible capacitation status. Regarding this point, one should take into account that the number of ovulated oocytes is greater than is the final number of implanted embryos^50^. This implies that the number of total embryos that a sow can produce after an ovulation does not depend on the total number of ovulated oocytes, but rather on the number of oocytes that are optimally fecundated. Following this rationale, photo-stimulated sperm would have a greater fertilising ability, thus increasing the number of fully functional embryos yielded.

In conclusion, the present work has demonstrated for the first time that photo-stimulation of liquid-stored boar semen increases sperm fertilising ability and reproductive performance. While farms would benefit from the use of photo-stimulation, research is required to address how red-based light exerts its action upon sperm. In addition, our data warrant further research in other mammalian species (including human). However, precise studies are required to elucidate optimal photo-stimulation conditions (including wavelength and time of exposure) in each species.

## Methods

### Suppliers

All reagents were of analytical grade and were purchased from Boehringer-Mannheim (Mannheim, Germany), Merck (Darmstadt, Germany) and Sigma-Aldrich (Saint Louis, MO, USA). As far as fluorochromes are concerned, and unless otherwise stated, all were purchased from Molecular Probes (Invitrogen; Eugene, Oregon, USA) and were previously resuspended with dimethyl sulfoxide (Sigma-Aldrich).

### Semen samples

In this study, boars were not handled by our team, but rather semen samples were obtained from a local farm (Servicios Genéticos Porcinos, S.L.; Roda de Ter, Barcelona, Spain). Thus, all ejaculates were initially intended for AI purposes, and were merely bought for conducting our experiments. In spite of this, and even not required, as we did not manipulate any boar, the experimental protocol was specifically approved by the Ethics Committee of our institution (Bioethics Commission, Autonomous University of Barcelona, Cerdanyola del Vallès, Spain). Furthermore, the handling of boars by the local farm was performed in accordance with EU Directive 2010/63/EU for animal experiments and the Animal Welfare Law issued by the Regional Government of Catalonia (Generalitat de Catalunya, Spain).

For the experiments conducted in our laboratory (i.e., evaluation of sperm resistance and achievement of ‘*in vitro*’ capacitation following different photo-stimulation treatments), a total of twelve different ejaculates, each coming from a separate boar, were used. Therefore, up to different 12 boars (all of proven fertility) were involved in those experiments. Boar semen was manually collected by the gloved-hand method. The sperm-rich fraction was diluted with a commercial extender for liquid-stored semen (MR-A Extender; Kubus, S.A.; Majadahonda, Spain), split up into seminal doses of 60 mL (2 × 10^9^ sperm per dose), and cooled down to 17 °C. Three seminal doses per ejaculate were randomly chosen and immediately transported in a portable refrigerator at 17 °C for approximately 45 min, which was the time required to arrive at the laboratory. Upon arrival, seminal doses coming from the same ejaculate were pooled. All experiments described below were thus repeated twelve times, using a total of 36 seminal doses coming from twelve boar ejaculates. Prior to starting any experiment, we confirmed that all seminal doses presented an acceptable sperm quality, the percentages of viable and total motile sperm (evaluated as described below) being higher than 85% and 80%, respectively^48^.

### Photo-stimulation procedures

Photo-stimulation was carried out in 1.7-mL reaction tubes, made up of highly transparent plastic (GenIUL, S.A.; Terrassa, Spain), which contained 1 mL of semen sample. Tubes were placed into a programmable photo-activation system (PhastBlue; GenIUL, S.A.). In this system, each tube is in contact with a triple LED setup that emits in the red-window wavelength (wavelength window: 620 nm–630 nm). This system is equipped with a software by which both the intensity and the time of emission of each LED can be graduated.

Preliminary experiments conducted in our laboratory indicated that exposing boar semen to single periods of red light exposure (ranging from 5 min to 20 min) had no clear effects on sperm quality. Taking this into account, it was subsequently decided to expose semen to sequential light-exposure periods (L-phase) separated by a darkness period (D-phase). Up to three photo-stimulation patterns were tested: (i) a first L-phase of 10 min, followed by a D-phase of 10 min and a final L-phase of 10 min (10-10-10 pattern; Photo-stimulation Procedure #1); (ii) a first L-phase of 15 min, followed by a D-phase of 10 min and a final L-phase of 15 min (15-10-15 pattern; Photo-stimulation Procedure #2); and (iii) a first L-phase of 20 min, followed by a D-phase of 10 min and a final L-phase of other 20 (20-10-20 pattern; Photo-stimulation Procedure #3).

The light intensity was kept at 100% in all cases and did not affect the temperature of samples. Internal temperature was determined by using an internal electronic thermometer structurally incorporated into the device. Therefore, there were no heating-related effects of photo-stimulation on sperm function. In addition, photo-stimulation procedures were performed at 16 °C by maintaining the equipment in a temperature-controlled room. As a control, a non-photo-stimulated 1 mL-aliquot of the same sample was placed in another reaction tube and stored at 16 °C. Thus, the environmental temperature for both non-photo-stimulated and photo-stimulated samples was the same. Reaction tubes utilised in control samples were the same (same volume and material) as those used for light treatments. Semen samples were subsequently subjected to either incubation at 37 °C for 90 min or ‘*in vitro*’ capacitation followed by progesterone-induced acrosome exocytosis immediately after photo-stimulation treatment.

### Evaluation of photo-stimulation on sperm resistance at 37 °C

When stated, 1-mL aliquots of semen samples, either with or without a previous photo-stimulation procedure, were taken and placed into a temperature-graduated water bath (37 °C) in new reaction tubes. Samples were then incubated for 90 min, and sperm viability and acrosome integrity, motility, and mitochondrial membrane potential were evaluated at 0 min, 15 min, 30 min, 60 min and 90 min of incubation. Special care was taken in this procedure, and semen samples were completely covered by water throughout all of the incubation period.

### Evaluation of photo-stimulation on ‘*in vitro*’ capacitation and progesterone-induced acrosome exocytosis

For ‘*in vitro*’ capacitation (IVC) and progesterone-induced acrosome exocytosis (IVAE), semen samples were washed three times through centrifugation (600 × *g* at 16 °C for 5 min) and then resuspended with phosphate buffered saline (PBS). This series of washing steps allowed for the elimination of any traces of seminal plasma and commercial extender. After the last centrifugation, samples were resuspended in non-capacitating medium (NCM, Tyrode’s-modified medium, albumin- and bicarbonate-free), which was made up of 20 mM 4-(2-hydroxyethyl)-1-piperazineethanesulfonic acid (Hepes) buffer containing 112 mM NaCl, 3.1 mM KCl, 5 mM glucose, 21.7 mM L-lactate, 1 mM sodium pyruvate, 0.3 mM Na_2_HPO_4_, 0.4 mM MgSO_4_ and 4.5 mM CaCl_2_. The osmolarity was 304 ± 5 mOsm·Kg^−1^, and pH was adjusted to 7.4. After the last wash, the spermatozoa were resuspended in a capacitating medium (CM), which consisted of NCM supplemented with 5 mg·mL^−1^ of bovine serum albumin (BSA) and 36 mM NaHCO_3_, to a final concentration of 20 × 10^6^ spermatozoa·mL^−1^. Incubation in CM was performed in a Heracell^®^ 150 incubator (Heraeus Instruments GmbH; Osterode, Germany) at 38.5 °C and 5% CO_2_ for 4 h, as described previously^51^.

Following IVC, the induction of IVAE was carried out through incubation with progesterone, as described before^30^,^52^. Briefly, progesterone was added to reach a final concentration of 10 μg·mL^−1^ to boar sperm previously incubated in CM for 4 h at 38.5 °C and 5% CO_2_. After thoroughly mixing, spermatozoa were further incubated for one hour in the same conditions (i.e., 38.5 °C, 5% CO_2_ atmosphere). Sperm aliquots of 1.5 mL each were taken at 0 h and 4 h of IVC, and 60 min after the induction of IVAE (i.e. 5 h).

The evaluation of both IVC and IVAE was performed through the analysis of previously described IVC- and IVAE-linked parameters^30^,^31^,^53^. These parameters were the percentage of viable spermatozoa subjected to progesterone-induced acrosome exocytosis (true acrosome exocytosis), the mean values of kinetic parameters after evaluation using a computer-assisted sperm-analysis system (CASA), the changes in cell-membrane lipid disorder and mitochondrial membrane potential (MMP) through JC-1 staining. The last two analyses were performed using flow cytometry as detailed in a subsection below.

### Analysis of sperm viability and acrosome integrity

Sperm viability and acrosome integrity were analysed in the evaluation of both sperm resistance at 37 °C and IVC-IVAE using three different fluorochromes: Hoescht 33258, propidium iodide and trypsin-inhibitor from soybean (SBTI) conjugated with Alexa Fluor^®^488, as described in ref. 54. Briefly, an aliquot of sperm suspension was incubated with Hoescht 33258 at a final concentration of 15 μM for 10 min at 37 °C. The sperm was subsequently incubated with propidium iodide (final concentration: 12 μM) at 37 °C for 5 min. Following centrifugation at 600 × *g* for 10 min, the supernatant was discarded and the sperm pellet obtained was resuspended in 1 mL of CM without BSA and bicarbonate, and containing Alexa Fluor^®^ 488-conjugated SBTI (SBTI-AF488; final concentration: 15 μM). Samples were incubated at 37 °C for 20 min and then centrifuged at 600 × *g* for 12 min. The resultant supernatant was discarded, and the sperm pellet was resuspended in 1 mL of NCM. The sperm was immediately evaluated under a Zeiss Axioskop-40 fluorescence microscope (Karl Zeiss GmbH; Jena, Germany) with the appropriate filters. With this purpose, a 5-μL drop per replicate (three replicates per sample were evaluated) was deposited on a slide and covered by a coverslip. Percentages of viable spermatozoa exhibiting intact or altered acrosomes were determined by counting 100 spermatozoa in each replicate at 400 × magnification. The corresponding mean ± standard error of the mean (SEM) resulting from the three counts (replicates) was calculated per sample and time-point. While unaltered acrosomes were considered to be those that did not present SBTI-AF488 staining, those altered showed a very intense SBTI-AF488-staining. Therefore, percentages of viable sperm exhibiting an intact acrosome presented no PI-labelling and were devoid of SBTI-AF488 staining. Non-viable sperm showed an intense red staining at the head. In the case of the evaluation of IVC achievement and progesterone-induced IVAE, spermatozoa subjected to a true acrosome exocytosis were considered to be those viable ones that presented an intense, non-uniform SBTI-AF488 staining.

### Analysis of sperm motility

Analyses of sperm motility in both the evaluation of sperm resistance at 37 °C and the achievement of IVC and progesterone-induced IVAE were performed through a computer-assisted sperm-analysis (CASA) system (Integrated Sperm Analysis System V1.0; Proiser S.L.; Valencia, Spain). Prior to evaluation, all samples were warmed at 37 °C for 15 min in a water bath, and one 10-μL drop per sample was placed onto a warmed (37 °C) Neubauer chamber (Paul Marienfeld GmbH & Co. KG; Lauda-Konigshofen, Germany). Our CASA system was based upon the analysis of 25 consecutive, digitalised photographic images obtained from a single field at a magnification of 100 × (negative phase-contrast field). These 25 consecutive photographs were taken at a velocity of image-capturing of one photograph every 40 msec. Different fields were taken up to reaching a minimum of 1,000 spermatozoa per replicate. Three replicates per sample and time-point were evaluated, prior to calculating the corresponding mean ± SEM.

The sperm kinetic parameters obtained were those described in ref. 55: curvilinear velocity (VCL, μm·s^−1^), which was the mean path velocity of the sperm head along its actual trajectory; straight-line velocity (VSL, μm·s^−1^), which was the mean path velocity of the sperm head along a straight line from its first to its last position; average path velocity (VAP, μm·s^−1^), which was the mean velocity of the sperm head along its average trajectory; percentage of linearity (LIN, %), which was the quotient between VSL and VCL multiplied by 100; percentage of straightness (STR, %), which was the quotient between VSL and VAP multiplied by 100; percentage of oscillation (WOB, %), which was the quotient between VAP and VCL multiplied by 100; mean amplitude of lateral head displacement (ALH, μm), which was the mean value of the extreme side-to-side movement of the sperm head in each beat cycle; and frequency of head displacement (BCF, Hz), which was the frequency at which the actual sperm trajectory crossed the average path trajectory (Hz). Total motility was defined as the percentage of spermatozoa showing a VAP higher than 10 μm·s^−1^ and progressive motility was defined as the percentage of spermatozoa that showed a VAP > 45 μm·s^−1^. Settings used for the CASA system were as follows: range of area particles: 10 μm^2^–80 μm^2^; connectivity: a minimum of 11 images for all parameters.

### Flow cytometry analyses

Flow cytometry was used to evaluate mitochondrial membrane potential in the evaluation of both sperm resistance at 37 °C and in achievement of IVC and progesterone-induced IVAE, and sperm-membrane lipid disorder was analysed only following IVC and progesterone-induced IVAE. In this section, information regarding flow cytometry analyses is given according to the recommendations of the International Society for Advancement of Cytometry^56^.

Prior to each assessment and at each relevant time-point, sperm concentration was adjusted to 1 × 10^6^ spermatozoa·mL^−1^ in a final volume of 0.5 mL as in ref. 57. After adjusting sperm concentration, samples were stained with the appropriate combinations of fluorochromes following the protocols described below. Evaluations were conducted through a Cell Laboratory Quanta SC^TM^ cytometer (Beckman Coulter; Fullerton, CA, USA), after excitation of particles with an argon ion laser (488 nm) set at a power of 22 mW. Cell diameter/volume was directly measured employing the Coulter principle for volume assessment, which evaluates electronic volume (EV) rather than forward scatter (FS). This EV channel was periodically calibrated using 10-μm Flow-Check fluorospheres (Beckman Coulter) by positioning this size at channel 200 on the volume scale. A total of three different optical filters were used with the following characteristics: FL1 (green fluorescence; YO-PRO-1, JC-1 monomers): Dichroic/Splitter, DRLP: 550 nm, BP filter: 525 nm, detection width 505 nm–545 nm; FL2 (orange fluorescence; JC-1 aggregates): DRLP: 600 nm, BP filter: 575 nm, detection width: 560–590 nm); FL3 (red fluorescence: Merocyanine-540) LP filter: 670 nm, detection width: 655 nm–685 nm.

Sheath flow-rate was set at 4.17 μL·min^−1^ in all analyses, and EV and side-scatter (SS) were recorded in a linear mode (in EV *vs.* SS dot plots) for a minimum of 10,000 events per replicate^58^. Signals were logarithmically amplified and photomultiplier settings were adjusted to particular staining methods. The analyser threshold was adjusted in the EV channel to exclude cell aggregates (particle diameter > 12 μm) and subcellular debris (particle diameter < 7 μm). Sperm-specific events were positively gated upon EV and SS distributions. In some protocols, compensation was used to minimise spillover of green fluorescence into the red channel, as described below. Dot-plots (FL1 *vs.* FL3; FL2 *vs.* FL3) were analysed through Cell Lab Quanta^®^SC MPL Analysis Software (version 1.0; Beckman Coulter). Flow cytometry data were corrected according to the procedure described by Petrunkina *et al*.^59^, as stated at the end of this section. Each assessment per sample and parameter was repeated three times in independent tubes, prior to calculating the corresponding mean ± SEM.

### Analysis of mitochondrial membrane potential

Mitochondrial membrane potential (MMP) was determined following the protocol described by Huo *et al*.^60^. Briefly, samples were incubated with JC-1 (5,5′,6,6′-tetrachloro-1,1′,3,3′tetraethylbenzimidazolylcarbocyanine iodide (final concentration: 0.3 μM) at 38 °C for 30 min in the dark. Two different emission filters (FL-1 and FL-2) were used to distinguish two sperm populations: (1) spermatozoa with high MMP (JC-1 aggregates), and (2) spermatozoa with low MMP (JC-1 monomers; ref. 55). The percentage of spermatozoa with high MMP corresponded to the orange-stained spermatozoa, which appeared in the upper half of the diagram in FL1 vs. FL2 dot-plots. Data were not compensated.

### Analysis of membrane lipid changes (M-540/YO-PRO-1)

Membrane lipid changes were assessed through co-staining with Merocyanine-540 (M-540) and YO-PRO-1, as described in ref. 61. Spermatozoa stained with M-540 (M-540^+^) presented a high membrane lipid disorder. YO-PRO-1 stain indicated early and degenerative changes in sperm membrane permeability. Those sperm negative for YO-PRO-1 and positive for M-540 (YO-PRO-1^-^/M540^+^) were those that showed fluidity membrane changes compatible with the achievement of feasible IVC. For this reason, percentages of viable sperm exhibiting positive staining for M540 are shown as an indicator of the IVC achievement^44^.

Sperm samples were incubated at 38 °C in the dark for 10 min with M-540 and YO-PRO-1 at final concentrations of 2.6 μM and 25 nM, respectively. The fluorescence of M-540 was detected through FL-3, whereas that of YO-PRO-1 was detected using FL-1. Unstained and single-stained samples were used for setting the EV-gain, FL-1 and FL-3 PMT voltages. Data were not compensated, and results are shown as the proportions of those viable sperm that exhibited high membrane lipid disorder (M540^+^).

### Correction of cytometric data: identification of non-DNA-containing particles

Data from all cytometric assessments were corrected following the protocol described by Petrunkina *et al*.^53^. This procedure determines the percentage of non-DNA-containing particles (in our study, non-sperm particles) and avoids an overestimation of sperm particles. Briefly, 5 μL of each analysed sperm aliquot were diluted with 895 μL of milliQ^®^-distilled water. Samples were then stained with PI at a final concentration of 12 μM and incubated at 38 °C for 3 min. Percentages of alien particles (*f*) were used to correct the percentages of non-stained spermatozoa (q_1_) in each sample after analysis according to the following formula: q_1_′ = [(q_1_−*f*)/(100−*f*)] × 100, where q′_1_ was the percentage of non-stained spermatozoa in the first quadrant after correction.

### Evaluation of photo-stimulation on reproductive performance

A total of 1,320 multiparous sows from a breeding farm (Servicios Genéticos Porcinos, S.L.; Sant Sadurní d’Osormort, Barcelona, Spain) were used in the fertility trials. Sows, from Landrace and Large White breeds, were housed in climate-controlled buildings, fed with an adjusted diet and provided with water ‘ad libitum’. Insemination trials covered a one-year period, according to an insemination programming system of all-in/all-out production followed by the breeding farm. Following this program, sows were chosen randomly to be included in the study. The insemination program was carried out according to the management of sows at weaning. Detection of oestrus was monitored from two days post-weaning by inspection of the vulva for reddening and swelling and response to a male teaser. Confirmation of oestrus was performed after four and five days post-weaning by pressing on the sow’s back and determining the presence of the standing reflex. The time of oestrus onset was defined as the first time at which a sow revealed a back-pressure response^62^.

Sows were inseminated weekly, each insemination group was composed of 25–26 sows, and a total of 51 AI trials was conducted. From those, 45 insemination trials involved 26 sows, whereas the other 6 involved 25 sows. This implies that 1,320 sows were involved in the present study (Supplementary Table S1). Artificial insemination was post-cervical^63^ through a Magaplus S^®^ catheter (Magapor; Zaragoza, Spain). Sows were inseminated twice, with an interval of 12 h between both inseminations, with diluted seminal doses prepared as previously described (i.e. 2 × 10^9^ sperm per dose in doses of 60 mL). Seminal doses stored at 17 °C were used within 12 h of ejaculate collection. No pooled ejaculates were made and thus each AI-dose came from a single boar.

When stated, 15–16 sows of each insemination trial were inseminated as described above with control, 60-mL standard AI doses. The other 9–11 sows were inseminated with 60-mL AI doses that had been previously photo-stimulated (Supplementary Table S1). Photo-stimulation of AI doses was carried out through a specifically designed portable refrigerator (MaXipig^®^; GenIUL, S.A.) consisting of a series of red LEDs that were distributed to uniformly illuminate a maximum of twenty-five 60-mL AI doses. This refrigerator also included an air-based refrigeration system to avoid excessive heating of the chamber induced by LEDs. The system was programmed to perform the first photo-stimulation procedure, which was the one that yielded the best results in the evaluation of sperm function and survival (first L-phase of 10 min, followed by a D-phase of 10 min and a final L-phase of 10 min; 10-10-10 pattern). Between 9 and 11 doses were simultaneously photo-stimulated per replicate. Artificial insemination was performed within 10 min after photo-simulation.

Non-return rates to oestrus were assessed at 21-days post-insemination (NRR_21d_) with a male teaser, and pregnancy rates (PR_30d_) were evaluated after 30 days through ultrasonography (Echoscan T-100; Import-Vet, S.A.; Barcelona, Spain), as described in ref. 64. At parturition, farrowing rates (FR) were recorded together with litter sizes, and evaluated by total number of piglets born (TP), number of live-born piglets (LP) and number of stillborn piglets (SP).

### Statistical analyses

All data were analysed with IBM SPSS for Windows (Version 21.0; SPSS Inc.; Chicago, Illinois, USA), and they are presented as percentages and means ± standard error of the mean (SEM).

Data (x) were first checked for normality and homogeneity of variances (homocedasticity) through Shapiro-Wilk and Levene tests, respectively. When required, data on percentages were transformed through arcsin √x. NRR_21d_, PR_30d_ and FR were transformed through logit transformation (i.e. logit = ln(NRR_21d_/(1 - NRR_21d_)).

The effects of photo-stimulation treatments upon sperm parameters, both in the assessment of sperm resistance at 37 °C for 90 min and that of IVC-IVAE achievement were evaluated separately through a mixed linear model (i.e. with repeated measures) followed by the post-hoc Sidak test, with incubation time as the intra-subject factor, photo-stimulation treatment as the fixed-effects factor and the boar as random-effects factor.

The effects of Photo-stimulation Treatment #1 (i.e., 10-10-10 pattern) upon reproductive performance parameters (i.e., logit-transformed NRR_21d_, PR_30d_ and FR, and TP, LP and SP were tested with a *t*-test and exact Fisher’s test (control *vs.* photo-stimulation treatment).

Each ejaculate was considered as an independent observation, and the minimal level of significance was set at *P* < 0.05 in all statistical analyses.

## Additional Information

**How to cite this article**: Yeste, M. *et al*. Specific LED-based red light photo-stimulation procedures improve overall sperm function and reproductive performance of boar ejaculates. *Sci. Rep.* **6**, 22569; doi: 10.1038/srep22569 (2016).

## Supplementary Material

Supplementary Information

## Figures and Tables

**Table 1:** Motility parameters of boar sperm subjected to separate photo-stimulation procedures and subsequently incubated at 37 °C for 90 min.

	Time	VCL (μm·s^−1^)	VSL (μm·s^−1^)	VAP (μm·s^−1^)	LIN (%)	STR (%)	WOB (%)	ALH (μm)	BCF (Hz)
Control	0 min	62.6 ± 3.4^a^	25.5 ± 1.6^a^	38.6 ± 2.1^a^	42.4 ± 2.4^a^	65.7 ± 2.6^a^	61.7 ± 1.5^a^	2.7 ± 0.1^a^	7.8 ± 0.2^a^
	15 min	38.4 ± 2.5^b^	23.8 ± 1.0^a^	30.3 ± 1.3^b^	63.6 ± 2.1^b^	78.2 ± 1.7^b^	79.2 ± 1.8^b^	1.6 ± 0.1^b^	7.3 ± 0.2^a^
	30 min	37.3 ± 1.2^b^	26.2 ± 1.6^a^	30.2 ± 1.2^b^	71.5 ± 2.6^c^	85.3 ± 1.8^c^	81.7 ± 1.6^bc^	1.6 ± 0.1^b^	6.8±0.2^b^
	60 min	41.3 ± 1.9^b^	29.1 ± 1.5^a^	33.9 ± 1.7^b^	73.7 ± 2.0^c^	87.3 ± 1.7^c^	83.2 ± 1.4^c^	1.3 ± 0.1^b^	7.6 ± 0.3^a^
	90 min	42.3 ± 2.0^b^	27.3 ± 1.8^a^	33.5 ± 2.1^b^	64.7 ± 3.7^b^	76.4 ± 2.7^b^	80.9 ± 2.7^bc^	1.4 ± 0.1^b^	7.2 ± 0.2^a^
Procedure #1 (10-10-10)	0 min	49.6 ± 2.3ª^*^	21.6 ± 1.7^a^	31.2 ± 1.5^a*^	46.4 ± 2.4^a^	71.8 ± 3.0^b^	63.2 ± 1.3^a^	2.1 ± 0.1ª*	9.2 ± 0.3^a*^
	15 min	37.5 ± 2.4^b^	21.1 ± 1.5^a^	27.6 ± 1.9^a^	61.9 ± 2.1^b^	76.5 ± 1.2^b^	79.2 ± 1.2^b^	1.6 ± 0.1^b^	7.2 ± 0.3^b^
	30 min	44.3 ± 3.9ª^*^	34.4 ± 3.4^b*^	38.1 ± 3.0^b*^	79.0 ± 1.8^c*^	90.2 ± 1.5^c^	87.6 ± 1.1^c*^	1.6 ± 0.1^a^	7.2 ± 0.2^b^
	60 min	47.0 ± 3.0^a*^	32.2 ± 2.0^b^	38.0 ± 2.4^b*^	73.2 ± 1.9^c^	86.3 ± 1.4^c^	84.0 ± 1.1^c^	1.5 ± 0.1^b^	7.8 ± 0.2^b^
	90 min	47.6 ± 1.3^a*^	33.9 ± 1.4^b*^	38.9 ± 0.9^b*^	76.9 ± 1.1^c*^	83.7 ± 1.4^c*^	86.4 ± 2.5^c^	1.8 ± 0.1^a^	9.1 ± 0.3^a*^
Procedure #2 (15-10-15)	0 min	63.3 ± 3.6^a^	27.0 ± 2.4^a^	41.5 ± 2.3^a^	44.1 ± 3.0^a^	66.1 ± 2.9^a^	64.2 ± 1.3^a^	2.5 ± 0.2^a^	8.6 ± 0.4^a*^
	15 min	43.7 ± 3.9^bc^	24.5 ± 1.9^a^	31.8 ± 2.3^b^	60.2 ± 2.0^b^	76.2 ± 1.2^b^	77.2 ± 1.9^b^	2.0 ± 0.1^b*^	8.3 ± 0.3^a*^
	30 min	37.1 ± 2.0^b^	25.7 ± 1.1^a^	31.0 ± 1.3^b^	71.2 ± 1.1^c^	82.8 ± 1.3^c*^	84.3 ± 0.9^c^	1.5 ± 0.1^c^	6.5 ± 0.5^b^
	60 min	38.8 ± 1.1^b^	29.9 ± 1.7^a^	33.2 ± 1.5^b^	73.8 ± 1.4^c^	86.5 ± 1.3^d^	84.8 ± 1.2^c^	1.4 ± 0.1^c^	7.9 ± 0.3^a^
	90 min	49.1 ± 2.7^c*^	36.8 ± 2.9^b*^	39.5 ± 2.2^a*^	80.8 ± 3.6^d*^	92.8 ± 3.0^e*^	89.1 ± 2.0^d*^	1.7 ± 0.1^c^	9.6 ± 0.5^b*^
Procedure #3 (20-10-20)	0 min	66.3 ± 2.2^a^	23.9 ± 1.4^a^	36.6 ± 1.3^a^	43.1 ± 1.5^a^	62.4 ± 3.3^a^	61.1 ± 1.1^a^	2.5 ± 0.1^a^	8.7 ± 0.5^a*^
	15 min	40.3 ± 2.2^b^	29.9 ± 1.9^b*^	31.4 ± 1.8^b^	74.5 ± 2.2^b*^	86.7 ± 1.4^b*^	84.4 ± 2.5^b^	1.6 ± 0.1^b^	7.4 ± 0.2^b^
	30 min	35.9 ± 1.6^b^	23.8 ± 1.6^a^	27.9 ± 1.5^b^	69.5 ± 1.1^c^	84.3 ± 1.8^b^	79.7 ± 1.3^b^	1.6 ± 0.0^b^	6.6 ± 0.5^b^
	60 min	48.7 ± 2.0^c*^	22.0 ± 1.2^a*^	32.3 ± 1.8^b^	49.2 ± 3.4^d*^	70.7 ± 2.6^c*^	76.9 ± 2.6^bc*^	1.9 ± 0.1^c*^	7.4 ± 0.3^b^
	90 min	51.2 ± 2.1^c*^	30.8 ± 1.2^b^	38.3 ± 1.0^a*^	68.4 ± 1.9^c^	87.0 ± 1.9^b*^	74.8 ± 1.9^c*^	2.3 ± 0.1^c*^	6.8 ± 0.4^b^

Boar spermatozoa were subjected to separate photo-stimulation procedures and subsequent incubation at 37 °C as described in the Methods section. At the indicated times, samples were taken and CASA-obtained motility parameters were registered. Both motility parameters and statistical procedures have been described in the Methods section. Results are shown as mean ± SEM. for 12 separate experiments. Different superscript letters indicate significant (*P* < 0.05) differences when compared with the respective 0-min point. Asterisks indicate significant (*P* < 0.05) differences when compared with the respective Control value at the same time point.

**Table 2:** Motility parameters of boar sperm subjected to ‘*in vitro*’ capacitation and further *‘in vitro*’ progesterone-induced acrosome exocytosis after being treated with separate photo-stimulation procedures.

	Time	VCL (μm·s^−1^)	VSL (μm·s^−1^)	VAP (μm·s^−1^)	LIN (%)	STR (%)	WOB (%)	ALH (μm)	BCF (Hz)
Control	0 h	61.0 ± 3.7^a^	23.2 ± 2.2^a^	34.6 ± 2.8^a^	37.3 ± 1.7^a^	62.3 ± 1.8^a^	56.6 ± 1.1^a^	2.6 ± 0.2^a^	5.8 ± 0.5^a^
	4 h	69.2 ± 3.3^b^	32.1 ± 3.1^b^	46.8 ± 2.4^b^	59.9 ± 2.7^b^	76.0 ± 4.0^b^	61.8 ± 2.4^b^	2.3 ± 0.2^a^	6.5 ± 0.3^b^
	5 h	79.1 ± 6.1^c^	35.4 ± 2.3^b^	42.1 ± 2.9^b^	62.9 ± 3.8^b^	90.0 ± 3.2^c^	62.0 ± 3.5^b^	3.4 ± 0.3^b^	6.2 ± 0.2^b^
Procedure #1 (10-10-10)	0 h	70.8 ± 2.8^a*^	23.6 ± 2.7^a^	38.6 ± 2.8^a^	32.0 ± 2.7^a*^	56.2 ± 3.8^a^	52.7 ± 1.7^a^	3.3 ± 0.2^a*^	5.1 ± 0.6^a^
	4 h	99.9 ± 4.9^b*^	30.7 ± 3.6^b^	47.5 ± 3.8^b^	47.5 ± 3.8^b*^	68.3 ± 4.6^b^	54.7 ± 2.4^a^	3.0 ± 0.3^a*^	6.7 ± 0.3^b^
	5 h	93.3 ± 2.0^b*^	67.1 ± 3.5^c*^	83.2 ± 1.8^c*^	92.4 ± 5.9^c*^	86.9 ± 4.7^c^	84.1 ± 4.2^b*^	2.3 ± 0.0^b*^	8.6 ± 0.7^c*^
Procedure #2 (15-10-15)	0 h	63.3 ± 2.8^a^	23.5 ± 2.5^a^	34.0 ± 2.6^a^	38.2 ± 2.0^a^	63.3 ± 2.0^a^	55.8 ± 2.1^a^	2.6 ± 0.2^a^	5.5 ± 0.2^a^
	4 h	81.5 ± 3.1^b*^	30.2 ± 2.6^b^	46.2 ± 2.5^b^	51.0 ± 5.0^b^	81.5 ± 4.9^b^	56.9 ± 4.0^a^	2.5 ± 0.1^a^	6.6 ± 0.4^b^
	5 h	61.2 ± 3.4^a*^	30.9 ± 2.8^b^	48.2 ± 3.5^b^	70.7 ± 3.2^b^	92.6 ± 2.7^c^	69.7 ± 3.5^b^	2.2 ± 0.1^b*^	6.7 ± 0.3^b^
Procedure #3 (20-10-20)	0 h	62.6 ± 3.6^a^	23.1 ± 2.6^a^	38.4 ± 2.9^a^	39.1 ± 2.6^a^	57.1 ± 3.6^a^	52.1 ± 2.6^a^	3.3 ± 0.2^a*^	5.1 ± 0.6^a^
	4 h	56.8 ± 3.5^b*^	24.8 ± 3.4^a*^	36.1 ± 2.9^a*^	43.2 ± 4.6^a*^	65.2 ± 4.1^b*^	60.6 ± 2.9^b^	2.5 ± 0.1^b^	6.3 ± 0.5^b^
	5 h	49.4 ± 4.0^c*^	14.9 ± 1.2^b*^	27.0 ± 2.7^b*^	33.0 ± 1.1^b*^	55.7 ± 3.1^a*^	54.2 ± 1.8^a*^	2.3 ± 0.2^b*^	6.9 ± 0.4^b^

Boar spermatozoa were subjected to separate photo-stimulation procedures and subsequent ‘*in vitro*’ capacitation and further progesterone-induced ‘*in vitro*’ acrosome exocytosis as described in the Methods section. At the indicated times, samples were taken and CASA-obtained motility parameters were registered. Both motility parameters and statistical procedures have been described in the Methods section. Results are shown as mean ± SEM for 12 separate experiments. Different superscript letters indicate significant (*P* < 0.05) differences when compared with the respective 0-h point. Asterisks indicate significant (*P* < 0.05) differences when compared with the respective Control value at the same time point 0 h. Values of sperm taken at 0 h of incubation in the capacitation medium (CM). 4 h: Values of sperm taken at 4 h of incubation in the CM. 5 h: Values of sperm taken after 4 h of IVC plus 60 min of the progesterone-induced IVAE.

**Table 3:** ‘*In vivo*’ fertility parameters of boar sperm ejaculates subjected to a previous photo-stimulation procedure.

Treatment	N	Farrowing rate (%)	Total piglets at parturition	Live-born piglets at parturition
Control	800	83.7	13.5 ± 0.2	12.7 ± 0.2
Photo-stimulated	520	88.1^*^	14.9 ± 0.3^*^	14.0 ± 0.2^*^

Sows were inseminated with AI doses subjected to a previous photo-stimulation procedure as described in the Methods section. Afterwards, main ‘*in vivo*’ fertility parameters were recorded as described in the Methods section. Asterisks indicate significant (*P* < 0.05) differences when compared with the Control group. Prolificacy data are given as mean ± SEM.

## References

[b1] Rodríguez-Gil J. E. & Estrada E. Artificial insemination in boar reproduction, in Boar Reproduction (eds Bonet S. .) Ch. 12, 589–608 (Springer, 2013).

[b2] Knox R. V. Artificial insemination in pigs today. Theriogenology 85, 83–93 (2016). 26253434 10.1016/j.theriogenology.2015.07.009

[b3] Huang Y. H., Lo L. L., Liu S. H. & Yang T. S. Age-related changes in semen quality characteristics and expectations of reproductive longevity in Duroc boars. Anim. Sci. J. 81, 432–437 (2010). 20662811 10.1111/j.1740-0929.2010.00753.x

[b4] Smital J., DeSousa L. L. & Mohsen A. Differences among breeds and manifestation of heterosis in AI boar sperm output. Anim. Reprod. Sci. 80, 121–130 (2004). 15036521 10.1016/S0378-4320(03)00142-8

[b5] Clark S. G., Schaeffer D. J. & Althouse G. C. B-mode ultrasonic evaluation of paired testicular diameter of mature boars in relation to average total sperm numbers. Theriogenology 60, 1011–1023 (2003). 12935842 10.1016/s0093-691x(03)00127-4

[b6] Leahy T. & Gadella B. M. Sperm surface changes and physiological consequences induced by sperm handling and storage. Reproduction 142, 759–778 (2011). 21964828 10.1530/REP-11-0310

[b7] Yeste M. . A diet supplemented with L-carnitine improves the sperm quality of Pietrain but not Duroc and Large White boars when photoperiod and temperature increase. Theriogenology 73, 577–586 (2010). 20022096 10.1016/j.theriogenology.2009.10.013

[b8] Yeste M., Barrera X., Coll D. & Bonet S. The effects on boar sperm quality of dietary supplementation with omega-3 polyunsaturated fatty acids differ among porcine breeds. Theriogenology 76, 184–196 (2011). 21458051 10.1016/j.theriogenology.2011.01.032

[b9] Kunavongkrit A., Suriyasomboon A., Lundeheim N., Heard T. & Einarsson S. Management and sperm production of boars under differing environmental conditions. Theriogenology 63, 657–667 (2005). 15626423 10.1016/j.theriogenology.2004.09.039

[b10] Pruneda A. . Effects of a high semen-collection frequency on the quality of sperm from ejaculates and from six epididymal regions in boars. Theriogenology 63, 2219–2232 (2005). 15826685 10.1016/j.theriogenology.2004.10.009

[b11] Ciereszko A., Ottobre J. S. & Glogowsdi J. Effects of season and breed on sperm acrosin activity and semen quality of boars. Anim. Reprod. Sci. 64, 89–96 (2000). 11078969 10.1016/s0378-4320(00)00194-9

[b12] Almond P. K. & Bilkei G. Seasonal infertility in large pig production units in an Eastern-European climate. Aust. Vet. J. 83, 344–346 (2005). 15986910 10.1111/j.1751-0813.2005.tb15627.x

[b13] Zasiadcyzk L., Fraser L., Kordan W. & Wasilewska K. Individual and seasonal variations in the quality of fractionated boar ejaculates. Theriogenology 83, 1287–1303 (2015). 25724288 10.1016/j.theriogenology.2015.01.015

[b14] Sancho S. . Semen quality of postpubertal boars during increasing and decreasing natural photoperiods. Theriogenology 62, 1271–1282 (2004). 15325554 10.1016/j.theriogenology.2004.01.003

[b15] Knecht D., Srodon S., Szulc K. & Duzinski K. The effect of photoperiod on selected parameters of boar semen. Livest. Sci. 157, 364–371 (2013).

[b16] Wegner K., Lambertz C., Daş G., Reiner G. & Gauly M. Climatic effects on sow fertility and piglet survival under influence of a moderate climate. Animal 8, 1526–1533 (2014). 24846319 10.1017/S1751731114001219

[b17] Iida R. & Koketsu Y. Interactions between climatic and production factors on returns of female pigs to service during summer in Japanese commercial breeding herds. Theriogenology 80, 487–493 (2013). 23756040 10.1016/j.theriogenology.2013.05.011

[b18] Rivera M. M., Quintero-Moreno A., Barrera X., Rigau T. & Rodríguez-Gil J. E. Effects of constant, 9-hours and 16-hours light cycles on sperm quality, semen storage ability and motile sperm subpopulations structure of boar semen. Reprod. Domest. Anim. 41, 386–393 (2006). 16984343 10.1111/j.1439-0531.2006.00677.x

[b19] Abdel-Salam Z. & Harith M. A. Laser researches on livestock semen and oocytes: a brief review. J. Adv. Res. 6, 311–317 (2015). 26257928 10.1016/j.jare.2014.11.006 PMC4522585

[b20] Lubart R., Friedmann H., Levinshal T., Lavi R. & Breibart H. Effect of light on calcium transport in bull sperm cells. J. Photochem. Photobiol. B 15, 337–341 (1992). 1432397 10.1016/1011-1344(92)85139-l

[b21] Cohen N., Lubart R., Rubinstein S. & Breitbart H. Light irradiation of mouse spermatozoa: stimulation of *in vitro* fertilization and calcium signals. J. Photochem. Photobiol. B 68, 407–413 (1998). 9747596

[b22] Zan-Bar T. . Influence of visible light and UV radiation on motility and fertility of mammalian and fish sperm. Photomed. Laser. Surg. 23, 549–555 (2005). 16356145 10.1089/pho.2005.23.549

[b23] Shahar S. . Light-mediated activation reveals a key role for protein kinase A and sarcoma protein kinase in the development of sperm hyper-activated motility. Hum. Reprod. 26, 2274–2282 (2011). 21771771 10.1093/humrep/der232

[b24] Corral-Baqués M. I., Rigau T., Rivera M., Rodríguez. J. E. & Rigau J. Effect of 655-nm diode laser on dog sperm motility. Lasers Med. Sci. 20, 28–34 (2005). 15838719 10.1007/s10103-005-0332-3

[b25] Corral-Baqués M. I., Rivera M. M., Rigau T., Rodríguez-Gil J. E. & Rigau J. The effect of low-level laser irradiation on dog spermatozoa motility is dependent on laser output power. Lasers Med. Sci. 24, 703–713 (2009). 18787758 10.1007/s10103-008-0606-7

[b26] Iaffaldano N., Meluzzi A., Manchisi A. & Passarella S. Improvement of stored turkey semen quality as a result of He-Ne laser irradiation. Anim. Reprod. Sci. 85, 317–325 (2005). 15581514 10.1016/j.anireprosci.2004.04.043

[b27] Iaffaldano N. . The irradiation of rabbit sperm cells with He–Ne laser prevents the *in vitro* liquid storage dependent damage. Anim. Reprod. Sci. 119, 123–129 (2010). 19932573 10.1016/j.anireprosci.2009.10.005

[b28] Iaffaldano N. . The post-thaw irradiation of avian spermatozoa with He–Ne laser differently affects chicken, pheasant and turkey sperm quality. Anim. Reprod. Sci. 142, 168–172 (2013). 24125852 10.1016/j.anireprosci.2013.09.010

[b29] García-Herreros M. . Boar sperm velocity and motility patterns under capacitating and non-capacitating incubation conditions. Theriogenology 63, 795–805 (2005). 15629798 10.1016/j.theriogenology.2004.05.003

[b30] Ramió L. . Dynamics of motile-sperm subpopulation structure in boar ejaculates subjected to “*in vitro*” capacitation and further “*in vitro*” acrosome reaction. Theriogenology 69, 501–512 (2008). 18068222 10.1016/j.theriogenology.2007.10.021

[b31] Ramió-Lluch L. . “*In vitro*” capacitation and acrosome reaction are concomitant with specific changes in mitochondrial activity in boar sperm: evidence for a nucleated mitochondrial activation and for the existence of a capacitation-sensitive subpopulational structure. Reprod. Domest. Anim. 46, 664–673 (2011). 21121968 10.1111/j.1439-0531.2010.01725.x

[b32] Yazdi S. R. . Effect of 830-nm diode laser irradiation on human sperm motility. Lasers Med. Sci. 29, 97–104 (2014). 23407899 10.1007/s10103-013-1276-7

[b33] Abdel-Salam Z., Dessouki S. H. M., Abdel-Salam S. A. M., Ibrahim M. A. M. & Harith M. A. Green laser irradiation effects on buffalo semen. Theriogenology 75, 988–994 (2011). 21220155 10.1016/j.theriogenology.2010.11.005

[b34] Begum R., Powner M. B., Hudson N., Hogg C. & Jeffery G. Treatment with 670 nm Light Up Regulates Cytochrome C Oxidase Expression and Reduces Inflammation in an Age-Related Macular Degeneration Model. PLOS One 8, e57828 (2013) doi: 10.1371/journal.pone.0057828. 23469078 PMC3585189

[b35] Passarella S., Casamassima E., Molinari S. & Pastone D. Increase of proton electrochemical potential and ATP synthesis in rat liver mitochondria irradiated *in vitro* by He-Ne laser. FEBS Lett. 175, 95–99 (1984). 6479342 10.1016/0014-5793(84)80577-3

[b36] Wenbin Y. . Effects of laser radiation on Saanen buck’s sperm energy metabolism. Proc. 6^th^ Int. Conf. Goats. Beijing, China. May 5–11 (1996).

[b37] Ainscow E. K. & Brand M. D. Top-down control analysis of ATP turnover, glycolysis and oxidative phosphorylation in rat hepatocytes. Eur. J. Biochem. 263, 671–685 (1999). 10469130 10.1046/j.1432-1327.1999.00534.x

[b38] Demaurex N., Poburko D. & Frieden M. Regulation of plasma membrane calcium fluxes by mitochondria. Biochim. Biophys. Acta 1787, 1383–1394 (2009). 19161976 10.1016/j.bbabio.2008.12.012

[b39] Nguyen T. T. . Mitochondrial oxidative stress mediates high phosphate-induced secretory defects and apoptosis in insulin-secreting cells. Am. J. Physiol. Endocrinol. Metab. 308, 933–941. (2015). 10.1152/ajpendo.00009.2015 25852001

[b40] Aitken R. J. Free radicals, lipid peroxidation and sperm function. Reprod. Fertil. Dev. 7, 659–668 (1995). 8711202 10.1071/rd9950659

[b41] Liu Y., Fiskum G. & Schubert D. Generation of reactive oxygen species by the mitochondrial electron transport chain. J. Neurochem. 80, 780–787 (2002). 11948241 10.1046/j.0022-3042.2002.00744.x

[b42] Aitken R. J., Paterson M., Fisher H., Buckingham D. W. & VanDuim M. Redox regulation of tyrosine phosphorylation in human spermatozoa and its role in the control of human sperm function. J. Cell Sci. 108, 2017–2025 (1995). 7544800 10.1242/jcs.108.5.2017

[b43] Martínez-Pastor F. . Reactive oxygen species generators affect quality parameters and apoptosis markers differently in red deer spermatozoa. Reproduction 137, 225–235 (2009). 19028926 10.1530/REP-08-0357

[b44] Gangwar D. K. & Atreja S. K. Signalling Events and Associated Pathways Related to the Mammalian Sperm Capacitation. Reprod. Domest. Anim. 50, 705–711 (2015). 26294224 10.1111/rda.12541

[b45] Sarewicz M. & Osyczka A. Electronic connection between the quinone and cytochrome C redox pools and its role in regulation of mitochondrial electron transport and redox signaling. Physiol. Rev. 95, 219–243 (2015). 25540143 10.1152/physrev.00006.2014 PMC4281590

[b46] Guthrie H. D. & Welch G. R. Effects of reactive oxygen species on sperm function. Theriogenology 78, 1700–1708 (2012). 22704396 10.1016/j.theriogenology.2012.05.002

[b47] Guthrie H. D. & Welch G. R. Determination of intracellular reactive oxygen species and high mitochondrial membrane potential in Percoll-treated viable boar sperm using fluorescence-activated flow cytometry. J. Anim. Sci. 84, 2089–2100 (2006). 16864869 10.2527/jas.2005-766

[b48] Yeste M., Estrada E., Casas I., Bonet S. & Rodríguez-Gil J. E. Good and bad freezability boar ejaculates differ in the integrity of nucleoprotein structure after freeze-thawing but not in ROS levels. Theriogenology 79, 929–939 (2013). 23398739 10.1016/j.theriogenology.2013.01.008

[b49] Ramió-Lluch L. . Oligomycin A-induced inhibition of the mitochondrial ATP synthase activity suppresses boar sperm motility and “*in vitro*” capacitation achievement without modifying the overall sperm energy levels. Reprod. Fertil. Dev. 26, 883–897 (2014). 25319379 10.1071/RD13145

[b50] Wähner N. & Brüssow K. P. Biological potential of fecundity of sows. Biotech. Anim. Husban. 25, 523–533 (2009).

[b51] Ramió-Lluch L. . “*In vitro*” capacitation and subsequent acrosome reaction are related to changes in the expression and location of midpiece actin and mitofusin-2 in boar spermatozoa. Theriogenology 77, 979–988 (2012). 22192394 10.1016/j.theriogenology.2011.10.004

[b52] Jiménez I. . Changes in the distribution of lectin receptors during capacitation and acrosome reaction in boar spermatozoa. Theriogenology 59, 1171–1180 (2003). 12527065 10.1016/s0093-691x(02)01175-5

[b53] Yeste M. . Intracellular calcium movements of boar sperm during *in vitro* capacitation and subsequent acrosome exocytosis follows a multiple storage places, extracellular calcium-dependent model. Andrology 3, 729–747 (2015). 26097097 10.1111/andr.12054

[b54] Yeste M. . Hyaluronic acid delays boar sperm capacitation after 3 days of storage at 15 degrees C. Anim. Reprod. Sci. 109, 236–250 (2008). 18162335 10.1016/j.anireprosci.2007.11.003

[b55] Yeste M. . Boar spermatozoa and prostaglandin F2alpha. Quality of boar sperm after the addition of prostaglandin F2alpha to the short-term extender over cooling time. Anim. Reprod. Sci. 108, 180–195 (2008). 17897798 10.1016/j.anireprosci.2007.08.008

[b56] Lee J. A. . The minimum information about a flow cytometry experiment. Cytometry A 73, 926–930. 18752282 10.1002/cyto.a.20623 PMC2773297

[b57] Yeste M. . Reduced glutathione and procaine hydrochloride protect the nucleoprotein structure of boar spermatozoa during freeze-thawing by stabilising disulfide bonds. Reprod. Fertil. Dev. 25, 1036–1050 (2013). 23089308 10.1071/RD12230

[b58] Garner D. L. & Johnson L. A. Viability assessment of mammalian sperm using SYBR-14 and propidium iodide. Biol. Reprod. 53, 276–284 (1995). 7492679 10.1095/biolreprod53.2.276

[b59] Petrunkina A. M., Waberski D., Bollwein H. & Sieme H. Identifying non-sperm particles during flow cytometric physiological assessment: a simple approach. Theriogenology 73, 995–1000 (2010). 20171719 10.1016/j.theriogenology.2009.12.006

[b60] Huo L. J., Ma X. H. & Yang Z. M. Assessment of sperm viability, mitochondrial activity, capacitation and acrosome intactness in extended boar semen during long-term storage. Theriogenology 58, 1349–1360 (2002). 12387348 10.1016/s0093-691x(02)00953-6

[b61] Harrison R. A. P., Ashworth P. J. & Miller N. G. A. Bicarbonate/CO_2_, an effector of capacitation, induces a rapid and reversible change in the lipid architecture of boar sperm plasma membranes. Mol. Reprod. Dev. 45, 378–391 (1996). 8916050 10.1002/(SICI)1098-2795(199611)45:3<378::AID-MRD16>3.0.CO;2-V

[b62] Roberts P. K. & Bilkei G. Field experiences on post-cervical artificial insemination in the sow. Reprod. Domest. Anim. 40, 489–491 (2005). 16149957 10.1111/j.1439-0531.2005.00616.x

[b63] Watson P. F. & Behan J. R. Intrauterine insemination of sows with reduced sperm numbers: results of a commercially based field trial. Theriogenology 57, 1683–1693 (2002). 12035978 10.1016/s0093-691x(02)00648-9

[b64] Estrada E. . Supplementing cryopreservation media with reduced glutathione increases fertility and prolificacy of sows inseminated with frozen-thawed boar semen. Andrology 2, 88–99 (2014). 24123940 10.1111/j.2047-2927.2013.00144.x

