# Supplementary material for: Specific LED-based red light photo-stimulation procedures improve overall sperm function and reproductive performance of boar ejaculates
Source: Sci Rep. 2016 Mar 2;6:22569. doi: 10.1038/srep22569 (PMC4773850; doi:10.1038/srep22569)
Supplement: Supplementary Information [file srep22569-s1.doc]

**Specific LED-based red light photo-stimulation procedures improve overall sperm function and reproductive performance of boar ejaculates**

Marc Yeste^1, 2, *^, Francesc Codony^3^, Efrén Estrada^1^, Miquel Lleonart^3^, Sam Balasch^4^, Alejandro Peña^1^, Sergi Bonet^5^, Joan E. Rodríguez-Gil^1, *^

^1^Department of Animal Medicine and Surgery, Faculty of Veterinary Medicine. Autonomous University of Barcelona, E-08193 Bellaterra (Cerdanyola del Vallès), Barcelona, Spain.

^2^Nuffield Department of Obstetrics and Gynaecology, University of Oxford, Level 3, Women’s Centre, John Radcliffe Hospital, Headington, Oxford OX3 9DU, United Kingdom.

^3^GenIUL, Rambla de Sant Nebridi 22, E-08222 Terrassa, Spain.

^4^Servicios Genéticos Porcinos, S.L., E-08150 Les Masies de Roda, Spain.

^5^Biotechnology of Animal and Human Reproduction (TechnoSperm), Department of Biology, Institute of Food and Agricultural Technology, University of Girona, E-17003 Girona, Spain

**^*^Corresponding authors**

Marc Yeste, Nuffield Department of Obstetrics and Gynaecology, University of Oxford, Level 3, Women’s Centre, John Radcliffe Hospital, Headington, Oxford, OX3 9DU, United Kingdom. Tel: +44 (0)1865 782829; Fax: +44 (0)1865 769141. E-mail: [marc.yeste@obs-gyn.ox.ac.uk](mailto:marc.yeste@obs-gyn.ox.ac.uk)

Joan E. Rodríguez-Gil, Department of Animal Medicine and Surgery, Faculty of Veterinary Medicine. Autonomous University of Barcelona, E-08193 Bellaterra (Cerdanyola del Vallès), Barcelona, Spain. Phone: +34-935811045. E-mail: [juanenrique.rodriguez@uab.cat](mailto:juanenrique.rodriguez@uab.cat)

**Supplementary Table S1** Number of sows involved in Artificial Insemination (AI) trials.

| **Number of AI trials** | **AI with control sperm per trial** | **Total sows inseminated with control sperm** | **AI with photo-stimulated sperm per trial** | **Total sows inseminated with photo-stimulated sperm** | **AI sows per trial** | **Total sows** |
| --- | --- | --- | --- | --- | --- | --- |
| 2 | 16 | 32 | 9 | 18 | 25 | 50 |
| 4 | 15 | 60 | 10 | 40 | 25 | 100 |
| 33 | 16 | 528 | 10 | 330 | 26 | 858 |
| 12 | 15 | 180 | 11 | 132 | 26 | 312 |
| **51** |  | **800** |  | **520** |  | **1,320** |
